# Mapping potential pathogen profiling in cetacean blow: comparative insights from sequencing technologies

**DOI:** 10.1099/mgen.0.001773

**Published:** 2026-07-08

**Authors:** Johann Frederick Jaitner, Nicola Gambardella, Luís Afonso, Raul Valente, Maria Paola Tomasino, Ana Mafalda Correia, Massimiliano Rosso, Filipe Alves, Catarina Magalhães

**Affiliations:** 1 CIIMAR/CIMAR LA, Interdisciplinary Centre of Marine and Environmental Research, University of Porto, Terminal de Cruzeiros do Porto de Leixões, 4450-208, Matosinhos, Portugal; 2 MIN-Faculty – Department of Biology, University of Hamburg, 22609, Hamburg, Germany; 3 School of Medicine and Biomedical Sciences (ICBAS), University of Porto, Porto, Portugal; 4 FCUP – Department of Biology, Faculty of Sciences, University of Porto, 4169-007, Porto, Portugal; 5 NBFC - National Biodiversity Future Center, 90133, Palermo, Italy; 6 MARE, Aquatic Research Network, Regional Agency for the Development of Research, Technology and Innovation (ARDITI), Funchal, Madeira, Portugal; 7 Faculty of Life Sciences, University of Madeira, Funchal, Madeira, Portugal

**Keywords:** blow sampling, *Delphinus delphis*, exhale breath condensate, *Globicephala macrorhynchus*, metabarcoding, pathogen detection

## Abstract

Cetaceans play a critical role in marine ecosystems and function as sentinel species for detecting environmental perturbations, underscoring the importance of assessing their health for effective marine conservation. This study employed 16S rRNA gene sequencing to characterize the prokaryotic communities present in exhaled breath condensate (EBC) samples from cetaceans, utilizing both short-read (Illumina) and long-read (PacBio) sequencing platforms. Putative pathogenic taxa were identified using the Multiple Bacterial Pathogen Detection (MBPD) database. Substantial differences in microbial community composition were observed between sequencing approaches. The PacBio platform yielded 2,373 amplicon sequence variants (ASVs) spanning 30 bacterial phyla, with 614 ASVs identified as potential pathogens. In contrast, the Illumina dataset generated 350 ASVs across 17 phyla, of which 46 were flagged as potentially pathogenic. Discrepancies were also evident in diversity metrics: PacBio-derived profiles exhibited higher alpha diversity and produced beta diversity clustering patterns that corresponded with sample metadata, while Illumina-based profiles did not reveal meaningful clustering. Distinct EBC microbial signatures were identified for *Globicephala macrorhynchus* and *Delphinus delphis*, with clear differences from the surrounding seawater microbiota. These findings support the use of EBC as a non-invasive and informative tool for respiratory microbiome analysis in marine mammals. Notably, this study provides the first characterization of the respiratory microbiota in *D. delphis*, offering a valuable methodological baseline for future research into host–microbiome interactions, health assessment and putative pathogen monitoring in free-ranging cetacean populations, using non-invasive approaches.

**Impact Statement**

This study advances non-invasive respiratory microbiome analysis in cetaceans through comparative evaluation of short-read and long-read 16S rRNA sequencing platforms. By analysing exhaled breath condensate (EBC) samples, we show that platform choice has a substantial impact on microbial community characterization, with PacBio yielding greater taxonomic resolution, higher alpha diversity, more robust beta diversity clustering and enhanced detection of potential pathogens relative to Illumina. Integration of the Multiple Bacterial Pathogen Detection (MBPD) database enabled identification of putative respiratory pathogens, providing a starting point for pathogen surveillance in free-ranging populations. Distinct microbiota were found, and the first description of the respiratory microbiome in *Delphinus delphis* was provided. These results highlight the critical importance of long-read sequencing and refined bioinformatic workflows in accurately capturing host-associated microbial communities in free-ranging cetaceans. By establishing a methodological baseline for respiratory microbiome research in marine mammals, this work provides a foundation for future efforts to standardize protocols, improve detection sensitivity and integrate EBC-based microbiome profiling into broader health assessment and monitoring strategies.

## Data Summary

Genomic data collected during this study are publicly available in the European Nucleotide Archive (ENA) under the accession number PRJEB97412. Particularly, the samples included in this study are available at the following sample accession numbers: SAMEA120139533, SAMEA120139537, SAMEA120139540, SAMEA120139541, SAMEA120139546, SAMEA120139543, SAMEA120139544, SAMEA120139534, SAMEA120139535, SAMEA120139535, SAMEA120139540, SAMEA120139542, SAMEA120139544, SAMEA120139546, SAMEA120139547, SAMEA120139548, SAMEA120139549, SAMEA120139532, SAMEA120139545, SAMEA120139548, SAMEA120139549, SAMEA120139542, SAMEA120139536, SAMEA120139531, SAMEA120139532, SAMEA120139533, SAMEA120139534, SAMEA120139536, SAMEA120139537, SAMEA120139538, SAMEA120139543, SAMEA120139545, SAMEA120139531, SAMEA120139538, SAMEA120139539, SAMEA120139547, SAMEA120139539 and SAMEA120139541. All other data supporting our findings are available in the supplementary material.

## Introduction

Cetaceans (whales, dolphins and porpoises) play a crucial ecological role as ecosystem engineers, contributing to nutrient transport and the formation of high productivity areas [1]. In addition to their status as keystone and flagship species for marine conservation [2], cetaceans are also recognized as bioindicators of environmental health, serving as sentinel for habitat changes [3]. Studying the health status of these marine mammal populations is therefore essential through effective and preferably non-invasive monitoring methodologies.

The analysis of the blow, or the exhaled breath condensate (EBC), is an emerging monitoring tool in cetology. Recent studies suggest that the EBC can serve as a biomarker for respiratory health [4] through the characterization of its microbial community [5,15]. Since respiratory infections are a common cause of mortality in marine mammals (16,17), advancing these monitoring techniques is crucial. Moreover, blow microbiota in cetacean have been shown to be influenced by host behaviour and health status [18]. The EBC microbial analysis enables the identification of potential pathogens [9] and provides insight into the vulnerability of cetaceans by comparing their respiratory microbiota with that of the surrounding environment. This approach can also reveal species-specific micro-organisms [8,11, 14, 19]. Recent studies have highlighted differences in the airway microbiota across cetacean species [15] and geographical regions [5], emphasizing the need for local studies. In addition, population structure and sociality may influence microbial composition, suggesting transmissibility among individuals [15,20].

Among the commonly used genetic markers, the 16S rRNA gene is the most widely employed for prokaryotic (Bacteria and Archaea) community detection [5,7, 9], although other genes, such as the 12S, have also been explored [21]. The 16S rRNA gene has thus become particularly interesting in recent decades, being applied in different contexts to study prokaryotic communities, usually by targeting a subregion of this same marker in short-read sequencing [22]. Advances in high-throughput sequencing now allow for full-length 16S rRNA sequencing, significantly improving taxonomic resolution [23]. This is particularly important for distinguishing between pathogenic and non-pathogenic species or strains within the same genus. However, current methods have yet to achieve species-level resolution, limiting the ability to accurately assess the pathogenic potential of micro-organisms in cetacean respiratory health [5]. Consequently, there is a pressing need for more advanced tools capable of delivering accurate and comprehensive insights to generate the pathogenic profiles in different EBC samples.

This study aims to advance microbiome analysis in EBC samples by comparing different 16S rRNA gene sequencing methodologies. Both short-read sequencing (targeting the V4–V5 region) and long-read sequencing (targeting the whole 16S rRNA gene) were employed to characterize the EBC microbiome and identify potential pathogens in two delphinid species, a taxonomic group for which research on this topic remains limited. The V4–V5 region, recommended by the Earth Microbiome Project [24], has been proposed as a particularly effective marker for the study of marine prokaryotes in EBC [20]. With the broader objective of monitoring free-ranging cetacean populations, our findings contribute to refining pathogen detection in EBC samples.

## Methods

### Sampling and sample preparation

**Fig. 1.**
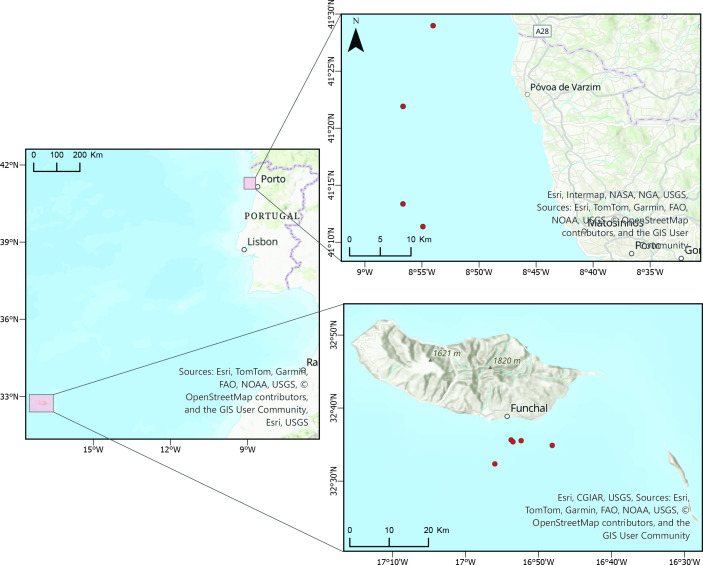
Location of the two study sites in the ENA. The red dots indicate the geographic locations where the EBC samples were collected.

In 2018, EBC samples from *Globicephala macrorhynchus* were collected in the southern waters of Madeira island (Portugal), and in 2021, from *Delphinus delphis* along the northern coast of mainland Portugal – both within the Eastern North Atlantic (ENA) region (Fig. 1, Table S1, available in the online Supplementary Material). Samples were obtained using an extendable 5 m aluminium pole with a PERFORMAgene™ PG-100 swab collection kit (DNA Genotek®) attached to its tip, which was held above the animal’s blowhole during exhalation. In 2018, alongside EBC samples, seawater samples were collected by dipping cotton swabs into the water and storing them in RNAlater at the time of collection. Additionally, as a control for seawater samples, 1 l of water was manually filtered through a Sterivex™ filter (Milipore) with a 0.22 µm pore size.

The DNA from EBC and seawater samples was isolated and purified using the QIAamp® DNA Mini and Blood Mini Handbook (Qiagen 2016), while the Sterivex samples were processed using DNeasy PowerWater Sterivex Kit (Qiagen 2019). Negative controls were included at the DNA extraction level. Specifically, a reagent-only negative control (i.e. no sample input) was processed alongside all samples using the QIAamp® DNA Mini and Blood Kit, following the manufacturer’s protocol. This approach is consistent with the recommendations of [25], who demonstrated that extraction blanks are essential for detecting kit-derived contaminants.

### Sequencing

The 16S rRNA gene was amplified via PCR in preparation for both short-read (Illumina) and long-read (PacBio) sequencing.

For Illumina sequencing, the 16S rRNA gene was amplified with the degenerate primer pair 515YF (5′-GTGYCAGCMGCCGCGGTA -3′) and Y926R-jed (5′-CCGYCAATTYMTTTRAGTTT-3′), targeting the hypervariable V4V5 region [26,27]. The initial PCR reaction included 12.5 ng of template DNA in a total volume of 25 µl. The PCR protocol consisted of 25 cycles of initial denaturation at 98 ˚°C for 30 s, denaturation at 98 °C for 10 s, blocking primer annealing at 70 °C for 30 s, annealing at 55 °C for 30 s and extension at 72 °C for 30 s, with a final extension at 72 °C for 4 min and 30 s. A second PCR was performed to add indexes and sequencing adapters to the target region, following the manufacturer’s recommendations (https://www.illumina.com/). Negative controls without template DNA were included in all PCR reactions. The final PCR products were one-step purified and normalized using SequalPrep 96-well plate kit (ThermoFisher Scientific, Waltham, USA), pooled, and paired-end sequenced (2×300 bp) on an Illumina MiSeq® sequencer using V3 chemistry at Genoinseq (Cantanhede, Portugal).

For PacBio sequencing, the 16S rRNA gene was amplified with the degenerate primer pair 27F (5′-AGRGTTYGATYMTGGCTCAG-3′) and 1492R (5′-RGYTACCTTGTTACGACTT-3′), targeting the full 16S rRNA gene (Paliy et al. 2009; Lane et al. 1991). Amplicon fragments were previously PCR-amplified in duplicate using separate template dilutions and a high-fidelity Phusion Plus polymerase. A single round of PCR was performed using full-length 16S rRNA primers. Negative controls without template DNA were included in all PCR reactions. PCR products were visually verified by running on a high-throughput Hamilton Nimbus Select robot using Coastal Genomics Analytical Gels. PCR reactions from the same samples were pooled into a single plate, cleaned and normalized using the high-throughput Charm Biotech Just-a-Plate 96-well Normalization Kit. PacBio samples were then pooled to make one library which was quantified fluorometrically before sequencing. Samples were then sequenced on a PacBio platform producing circular consensus sequencing (CCS) long reads.

Sequencing was performed by the Integrated Microbiome Resource (IMR), following their protocols (https://imr.bio/protocols.html).

### Data analysis

#### Upstream analysis

For Illumina data, 16S rRNA gene sequences were imported into QIIME2 [28], and DADA2 [29] was applied with settings specific for paired-end short reads. Forward and reverse reads were truncated at 280 and 270 bp, respectively, to retain only bases with an average Phred quality score above 30. A full summary of DADA2 statistics is available in Table S3. Amplicon sequence variants (ASVs) resulting from the analysis were then classified using a scikit-learn Naive-Bayes classifier trained on the silva database version 138.1 [30].

For PacBio, 16S rRNA gene sequences were also imported into QIIME2 [28], and DADA2 [29] was run with the specific option for CCS reads. Only reads with a length between 1,000 and 1,600 bp were kept. A table with the complete DADA2 statistics is available in Table S3. ASVs were also classified using a scikit-learn Naive-Bayes classifier trained on the same silva database as for Illumina data to maintain annotation consistency.

Rarefaction curves were generated to ensure sequencing depth was sufficient to capture the maximum possible microbial diversity (Fig. S1). Curves reached a plateau for both sequencing platforms, indicating adequate representation of the prokaryotic community. However, since some samples exhibited different sequencing depth, total sum scaling normalization has been deployed. This consisted of dividing the number of reads of each feature in a sample by the total sum of reads (of all features) in the sample and then multiplying by the desired number of reads [31].

#### Downstream analysis

Data analysis was conducted using Python (version 3.12.3) within the JupyterLab interface (version 4.0.11). The results from PacBio and Illumina were analysed separately but with similar scripts.

#### Relative abundance

Firstly, the feature and taxonomy tables were merged based on ASVs. To avoid skewing the community composition and diversity analyses, ASVs that could be assigned to chloroplasts at the genus level were removed for the subsequent steps. Next, the absolute abundance of each ASV was grouped by taxonomic level. Stacked bar plots were generated to visualize the percentage of relative abundances across samples. Colours in the bar plot and legend were manually assigned to ensure consistency between the Illumina and PacBio results.

#### Alpha and beta diversity

The metadata information was merged with the feature table and grouped by sample origin. Two commonly used metrics were applied to estimate alpha diversity values using the Scikit-Bio package version 0.6.0 (https://scikit.bio/index.html): Shannon Index [32] and the number of observed ASVs. The results of this analysis were presented in the form of a boxplot for both Illumina and PacBio datasets. A Wilcoxon signed-rank test followed by a Mann−Whitney U test was run to test for significant difference between sample origins (Tables S4– S7 and S10–S15).

A dendrogram based on Bray–Curtis dissimilarity [33] was generated to visualize the *β*-diversity among samples. After transposing the feature table, the ‘pdist’ function from the ‘spatial.distance’ module in the ‘scipy’ package (version 1.11.4) was used to calculate the pairwise distances between samples using the same metric. The resulting distance array was then utilized to perform hierarchical clustering using the ‘linkage’ function from the ‘cluster.hierarchy’ module, also from ‘scipy’, with the same metric. Finally, the ‘dendrogram’ function from the same module was used to visualize the clustering results. Analysis of similarities (ANOSIM) was run to test for significant grouping by sample origins (Tables S8 and S9).

### Potential pathogen identification and analysis

ASVs from Illumina and PacBio data were classified using the MBPD database [34] which contains a broad collection of known bacterial pathogens. ASVs were identified as putative pathogens if successfully classified by the Bayesian classifier trained on the database. Only ASVs classified at the strain level (presenting the prefix ‘s1_’) were kept for downstream analysis; unclassified ASVs were discarded. Since MBPD derives from the silva database, correct classification of the ASVs was tested by cross-validating MBPD-derived classifications with the silva taxonomy. The subsequent steps consisted of calculating the relative abundance, alpha and beta diversity of the potential pathogen community using the same methods applied to the whole microbial community.

## Results

### Overview of sequencing output and annotation

Thirteen EBC samples, eight from *G. macrohynchus* and five from *D. delphis*, were analysed.

Notably, the sequencing platforms employed in this study (Illumina and PacBio) yielded different descriptions of the microbial community. The Illumina platform produced an average of 54,512±31,413 sequences of 301 bp length, while PacBio yielded an average of 26,794±11,625 sequences with an average length of 1,483±9 bp. The PacBio platform resulted in 2,373 ASVs classified into 30 different phyla, with 614 ASVs flagged as potential pathogens. In contrast, Illumina sequencing resulted in 350 ASVs grouped into 17 phyla, of which 46 were identified as potential pathogens (Table S2).

### Prokaryotic community structure through Illumina and PacBio platforms

**Fig. 2.**
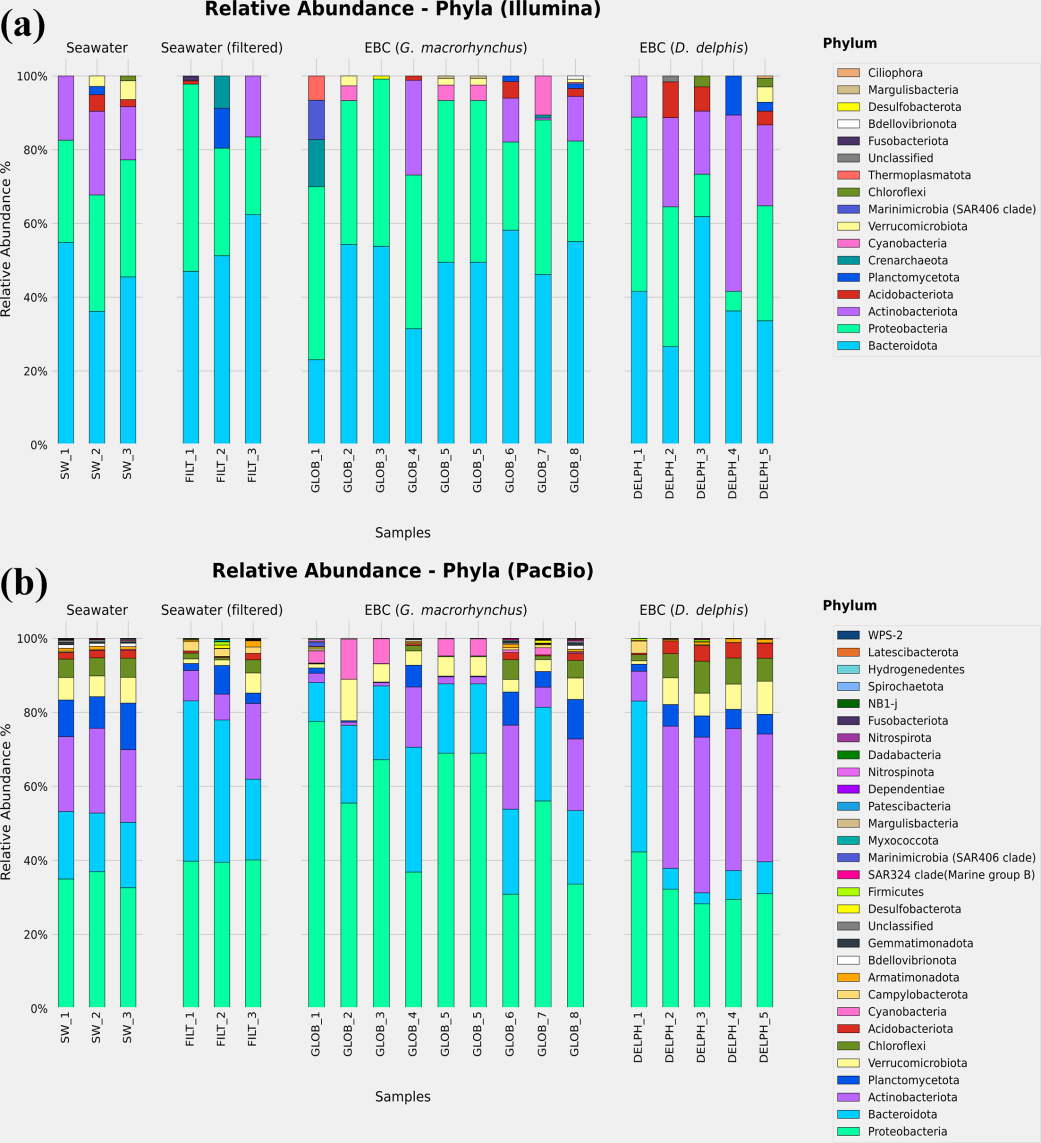
Barplot showing the relative abundance of the different phyla from the samples sequenced with Illumina (**a**) and PacBio (**b**), both based on the silva database. Bars represent different samples, and they were grouped by their sample origin. The coloured area represents the percentage of the respective phyla in the legend from the entire sample, sorted according to their relative abundance across all samples.

All sample origins sequenced with Illumina reported the presence of the following phyla: *Acidobacteriota* (unclassified genus, family Holophagaceae), *Actinobacteriota* (unclassified genus, family Sporichthyaceae), *Bacteroidota* (genera *Fluviicola*, *NS11* and *Algoriphagus*), *Planctomycetota* (genera *Blastopirellula*, *CL500*, *OM190* and uncultured from family *Gemmataceae*) and *Proteobacteria* (32 genera) (Figs 2a and S2A). Three of them, namely, *Proteobacteria*, *Bacteroidota* and *Actinobacteriota*, were the most abundant phyla in both Illumina and PacBio samples (Fig. 2) consisting of the genera *NS3A marine group*, *hgcI clade* and uncultured at the genus level (Fig. S2). *Bacteroidota* (mainly *Fluviicola* and *Sediminibacterium*) dominated in the Illumina samples, while *Proteobacteria* (mainly genera *Limnohabitans* and *Lentibacter*) was the most abundant phylum in the PacBio samples (Fig. 2b). Additionally, PacBio sequencing detected several phyla across all sample origins that were absent from the Illumina data. These uniquely detected phyla included *Armatimonadota* (genera *Fimbriimonadaceae* and *Armatimonas*), *Campylobacterota* (eight genera), *Dependentiae* (genera *Babeliales*, *Vermiphilaceae* and *UBA12409*), *Desulfobacterota* (ten genera), *Gemmatimonadota* (genus *Gemmatimonas* and sequences not classified at genus level) and *Patescibacteria* (five genera).

Some phyla were detected only in particular cetacean species. Illumina-sequenced EBC samples from *G. macrorhynchus* were uniquely characterized by *Bdellovibrionota* (*OM27_clade*), *Desulfobacterota* (unclassified at genus level), *Margulisbacteria* (genus *Margulisbacteria*), *Marinimicrobia (SAR406 clade*) and *Thermoplasmatota* (Marine group II) (Figs 2a and S2A). In contrast, the phylum *Ciliophora* (genus *Suctoria*), as well as unclassified prokaryotes, was exclusively found in the *D. delphis* blow samples obtained through Illumina (Fig. 2a). In *G. macrorhynchus* EBC samples sequenced with PacBio, the unique phyla were *Dadabacteria* (genus *Dadabacteriales*), *Nitrospinota* (genus *LS-NOB*) and *Spirochaetota* (genus *Spirochaeta*). No phyla were found to be exclusive to *D. delphis* EBC samples sequenced through PacBio.

For the swab seawater and filtered seawater samples, which served as environmental comparison samples for the *G. macrorhynchus* EBC samples, only filtered seawater contained a unique phylum (*Fusobacteriota*, genus *Leptotrichia*) among Illumina-sequenced samples. Of the 13 phyla identified in the EBC samples of *G. macrorhynchus*, seven were also present in both the seawater collected with a swab and in the filtered seawater samples. In contrast, *Bdellovibrionota* (*OM27 clade), Crenarchaeota* (genus *Nitrosarchaeum*)*, Desulfobacterota* (unclassified at genus level)*, Margulisbacteria* (genus *Margulisbacteria*)*, Marinimicrobia (SAR406 clade*) and *Thermoplasmatota* (Marine group II) were exclusively found in the EBC samples of *G. macrorhynchus* and were absent from the seawater collected with a swab. The same phyla were also undetected in filtered seawater samples, except for *Crenarchaeota*, while *Verrucomicrobiota* was absent.

In the PacBio-sequenced dataset, the phyla *Hydrogenedentes* (genus *Hydrogenedensaceae*) and *WPS-2* were unique to filtered seawater samples, whereas no unique phyla were identified in seawater samples collected with a swab. Of the 25 phyla identified in *G. macrorhynchus* blow samples, 20 were also present in the swab seawater samples and 19 in the filtered seawater samples. The five phyla absent from the swab seawater samples were *Dadabacteria* (genus *Dadabacteriales*)*, Marinimicrobia* (*SAR406 clade*), *NB1-j, Nitrospinota* (genus LS-NOB) and *Spirochaetota* (genus *Spirochaeta*). In the filtered seawater samples, *Dadabacteria*, *Margulisbacteria*, *Nitrospinota*, *SAR324 clade (Marine group B*) and *Spirochaetota* were not detected. Three phyla (*Dadabacteria*, *Nitrospinota* and *Spirochaetota*) were unique to *G. macrorhynchus* in the PacBio dataset, corresponding to the genera Dadabacteriales, *LS-NOB* and *Spirochaeta*, respectively. The *Thermoplasmatota* phylum (Marine group II), unique to *G. macrorhynchus*, was only detected in the Illumina dataset. *G. macrorhynchus*-specific phyla identified in the PacBio-sequenced EBC samples were not detected with Illumina.

Within the Illumina-sequenced *D. delphis* EBC samples, *Actinobacteriota* (unclassified at genus level), *Bacteroidota* (genus *Fluviicola*) and *Proteobacteria* (uncultured) were present in all five samples. *Proteobacteria* (genus *Methylopumilus*) was found in three individuals (DELPH_2, DELPH_3 and DELPH_5), *Chloroflexi* (SL56_marine_group) in two individuals (DELPH_3 and DELPH_5), as well as *Planctomycetota*, uncultured (DELPH_4 and DELPH_5). *Verrucomicrobia* (genus *Terrimicrobium*) was detected only in DELPH_5, along with the unique phylum *Ciliophora* (genus *Suctoria*). Unclassified organisms were found only in DELPH_2.

Among the five PacBio-sequenced *D. delphis* EBC samples, the phyla *Acidobacteriota* (genera *Vicinamibacteraceae* and marine group), *Actinobacteriota* (nine genera), *Armatimonadota* (genus *Armatimonas*), *Bacteroidota* (eight genera), *Chloroflexi* (SL56 marine group and TK10), *Planctomycetota* (genera CL500 and *Schlesneria*), *Proteobacteria* (22 genera) and *Verrucomicrobiota* (genus *Terrimicrobium*) were present in all. *Campylobacterota* and *Firmicutes* were absent in DELPH_2, *Patescibacteria* was missing in DELPH_5 and *Gemmatimonadota* was absent in DELPH_2. The phyla *Dependentiae*, *Desulfobacterota* and *SAR324* clade (Marine group B) were detected only in DELPH_2, DELPH_3 and DELPH_5. *Fusobacteriota* occurred only in DELPH_1 and *Latescibacterota* only in DELPH_3.

### Prokaryotic community diversity

**Fig. 3.**
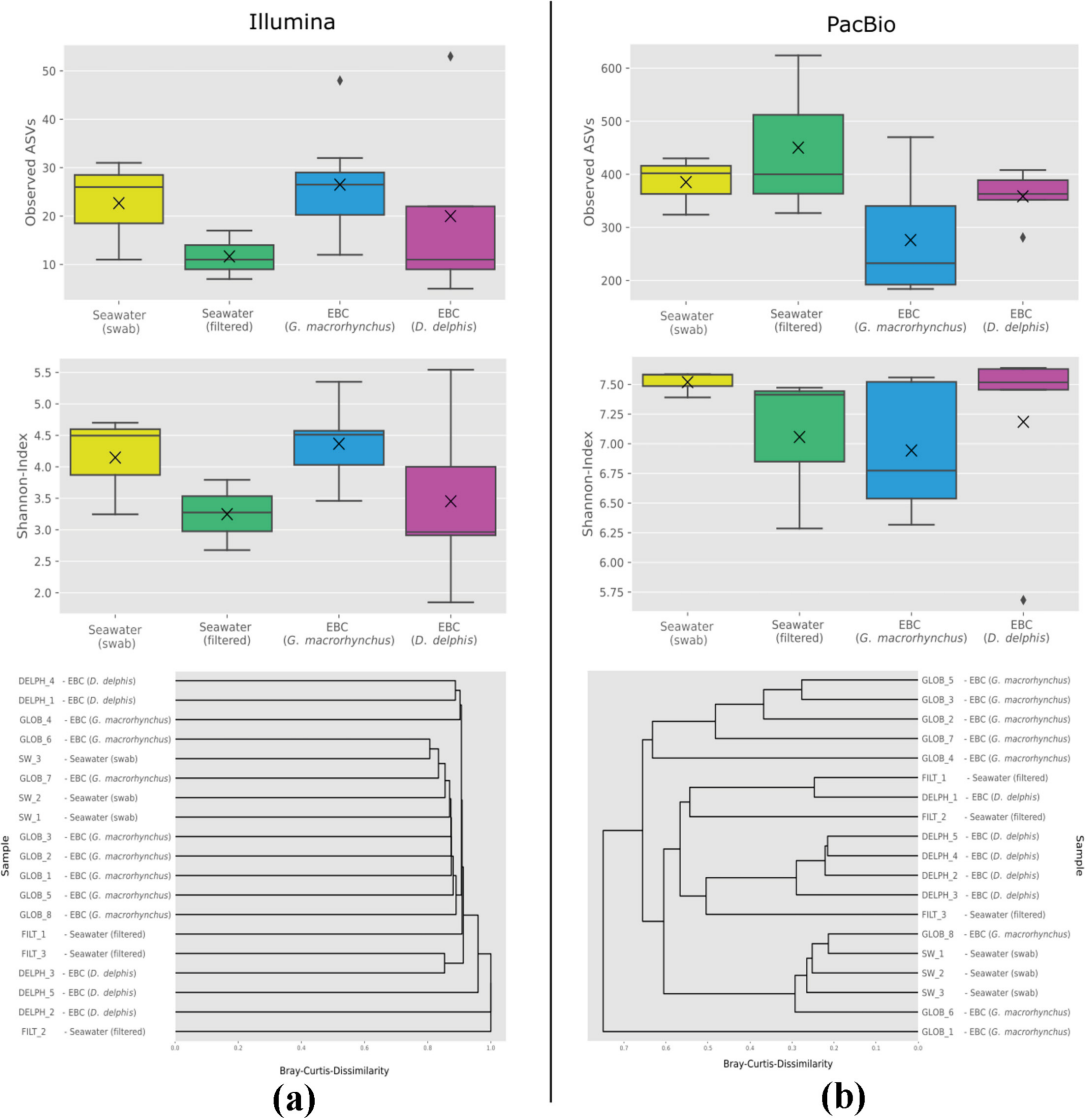
Boxplot diagram showing the alpha diversity for samples grouped according to their origin (top part), sequenced with Illumina (**a**) and PacBio (**b**). The alpha diversity was plotted on the y-axis using the number of observed ASVs and their values obtained by the calculation with the Shannon index. The mean value of the respective boxplots is labelled ‘X’. Dendrogram showing the hierarchical clustering of samples (bottom part) sequenced with Illumina (**a**) and PacBio (**b**) based on their Bray–Curtis dissimilarity of their general microbial community composition (indicated on the x-axis). The respective samples and their origin are shown on the y-axis.

To assess alpha diversity across the multiple samples, observed ASVs and the Shannon index were calculated for both sequencing methods (Fig. 3, top part). In the Illumina dataset, seawater (swab) and EBC (*G. macrorhynchus*) reported a higher diversity than seawater (filtered) and EBC (*D. delphis*) in both *α*-diversity metrics (Fig. 3a, top part). However, a Mann–Whitney U test between sample origins resulted in no significant difference (p-value >0.05, Table S6).

PacBio sequencing yielded 2,373 ASVs, resulting in higher observed ASVs and in the Shannon index values. Mann–Whitney U test run on *α-*diversity of the overall community between platforms reported significant difference between Illumina and PacBio with the former presenting significantly less diversity than the latter (p-value<0.05, Table S10). The same test returned no significant difference between sample origins (Table S10). In PacBio, the number of ASVs observed in all seawater samples was higher than in the EBC samples (Fig. 3b, top part). However, when considering the Shannon index, only the seawater samples collected with the swab reported higher diversity than the EBC samples (Fig. 3b, top part). *G. macrorhynchus* and *D. delphis* blow samples had similar diversity values independent from the metric considered (Fig. 3b, top part). Differences between sample origins were not significant (Mann–Whitney U test, p-value >0.05, Table S5).

*β*-Diversity analysis based on Bray–Curtis dissimilarity of the microbial composition in each sample revealed distinct clustering patterns among sample origins for both sequencing platforms (Fig. 3, bottom).

In the Illumina-based dendrogram (Fig. 3a, bottom part), no meaningful cluster was observed. However, ANOSIM on Illumina *β*-diversity of the overall community reported significant positive grouping by sample origin (*R*=0.304, p-value <0.05, Table S9).

The PacBio-based dendrogram (Fig. 3b, bottom part) revealed five clusters, largely corresponding to sample origins. Similarly to Illumina, ANOSIM reported significant positive grouping by sample origin for PacBio (*R*=0.366, p-value <0.05, Table S8). The first cluster consisted of *G. macrorhynchus* blow samples GLOB_2, GLOB_3, GLOB_5, GLOB_4 and GLOB_7. The other sample belonging to *G. macrorhynchus*, GLOB_1, formed a separate cluster due to its distinct microbial composition. The second cluster included *G. macrorhynchus* blow samples GLOB_6 and GLOB_8 alongside swab seawater samples, indicating a closer microbial relationship. The *D. delphis* EBC samples clustered together with FILT_3, except for DELPH_1, which formed a separate cluster together with the remaining filtered seawater samples.

### EBC profiles of potential prokaryotic pathogens

**Fig. 4.**
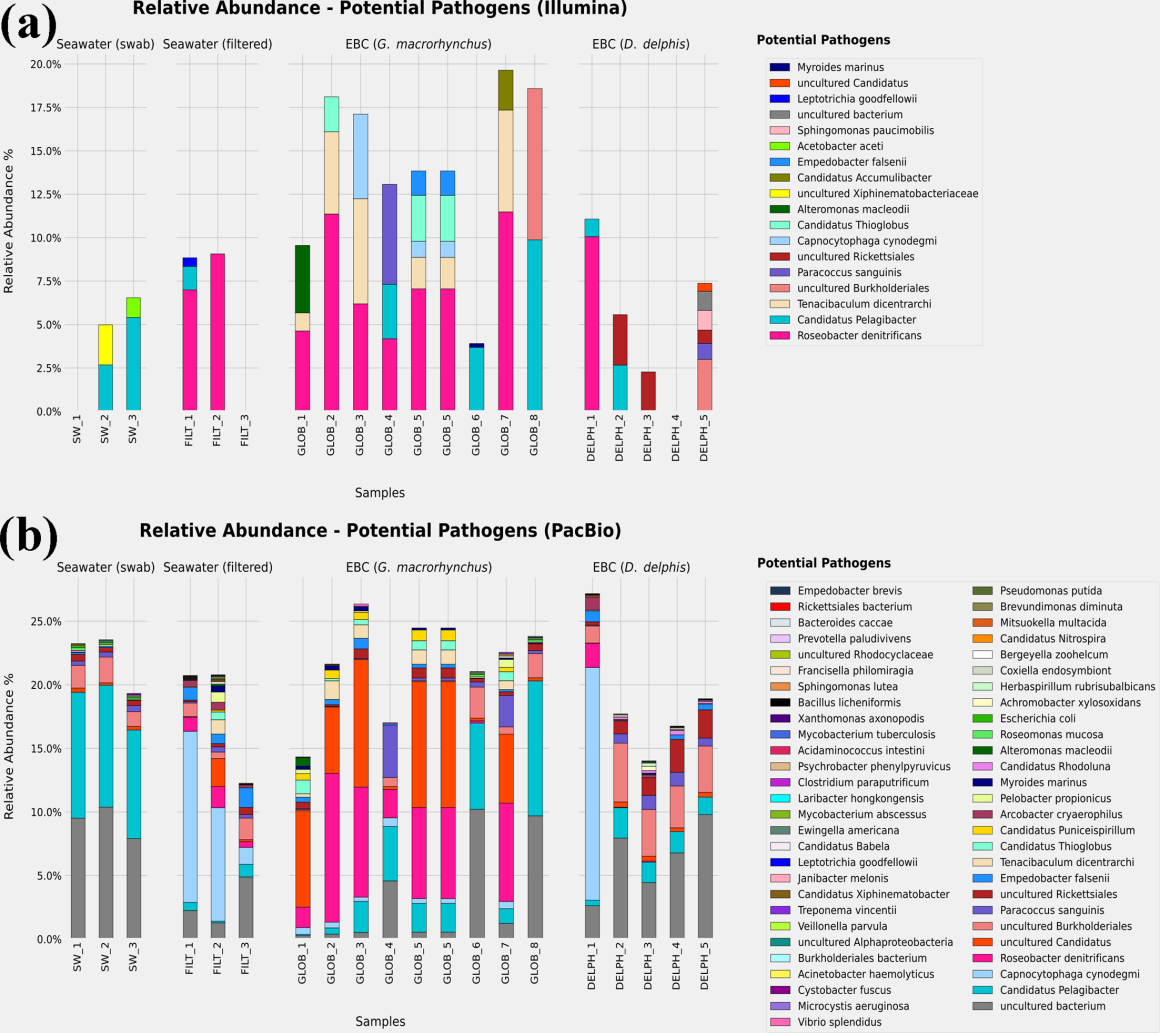
Stacked bar plot diagram showing the relative abundance of putative pathogens in the samples sequenced with Illumina (**a**) and PacBio (**b**). The potential pathogens identified are based on taxonomic matches between the silva and MBPD databases at the genus level. The bars/samples are grouped according to their sample origin. The coloured area represents the percentage of each respective putative pathogen (as indicated in the legend) in the total sample, sorted by their relative abundance across all samples.

Of the 350 ASVs obtained from Illumina sequencing, 46 were identified as potential pathogens based on the MBPD database. A total of 18 different putative pathogens were detected, distributed across various samples (Fig. 4a). No potential pathogens were found in samples SW_1 (seawater swab), DELPH_4 (*D. delphis*) and FILT_3 (filtered seawater). The relative abundance and putative pathogen composition varied between the individual samples and the sample origins. Overall, *Roseobacter denitrificans* constituted the largest proportion, being particularly abundant in the blow samples of *G. macrorhynchus*, while also appearing in one blow sample of *D. delphis* and in the seawater (filtered) samples (Fig. 4a). *Candidatus Pelagibacter*, which may have pathogenic activities [34], was detected in all sample origins, whereas some putative pathogens were found exclusively in a single sample origin. The following potential pathogens were exclusively found in the blow samples of *G. macrorhynchus: Alteromonas macleodii*, *Candidatus Accumulibacter*, *Candidatus Thioglobus*, *Capnocytophaga cynodegmi, Empedobacter falsenii*, *Myroides marinus* and *Tenacibaculum dicentrarchi*. Among these, *Tenacibaculum dicentrarchi* was present in multiple samples (GLOB_1, GLOB_2, GLOB_3, GLOB_5 and GLOB_7) with a notable proportion of the relative abundance, whereas others, such as *A. macleodii*, were detected in only a single sample (GLOB_1) (Fig. 4a). The potential pathogens *Acetobacter aceti* and *uncultured Xiphinematobacteriaceae* were exclusively found in seawater samples collected with swab, whereas *Leptotrichia goodfellowii* was only detected in seawater (filtered) samples. Among the 11 putative pathogens identified in the blow samples of *G. macrorhynchus*, one (*Candidatus Pelagibacter*) was also found in swab seawater, while two (*Candidatus Pelagibacter* and *R. denitrificans*) were also detected in filtered seawater (filtered) samples. The potential pathogens *Sphingomonas paucimobilis*, *uncultured Candidatus*, *uncultured Rickettsiales* and *uncultured bacterium* were exclusively found in the blow samples of *D. delphis*, specifically in sample DELPH_5.

Of the 2,373 ASVs obtained from PacBio sequencing, 614 were associated with potential pathogens, leading to a higher overall number of distinct putative pathogens (55) compared to the 18 identified through Illumina sequencing. The relative abundance of potential pathogens was also higher in the PacBio-sequenced samples compared to Illumina (Fig. 4). While putative pathogens’ proportions in the Illumina samples range from 0 to 18.5%, PacBio samples showed potential pathogens in every sample, with proportions fluctuating between 12.2 and 27.2%. The difference in putative pathogen detection between the two sequencing platforms resulted in Illumina reporting significantly lower values in terms of potential pathogen diversity (Mann–Whitney U test, p-value <0.05, Table S15). Uncultured putative pathogens such as *uncultured Burkholderiales*, *uncultured Candidatus*, *uncultured Rhodocyclaceae* and *uncultured bacteria* constitute a large proportion of potential pathogens (Fig. 4). Additionally, *Candidatus Pelagibacter*, which, like *uncultured Candidatus* and *uncultured bacteria*, was present in every single sample, reported high relative abundance. As observed in the Illumina-sequenced samples, in the PacBio dataset, the putative pathogen composition varied depending on the sample origin. Comparable abundances and distributions of *R. denitrificans* and *T. dicentrarchi* were observed in the *G. macrorhynchus* EBC samples sequenced with PacBio, mirroring the patterns found in those sequenced with Illumina. Additionally, samples GLOB_1, GLOB_2, GLOB_3, GLOB_5 and GLOB were characterized by a high proportion of uncultured *Candidatus*. Samples GLOB_6 and GLOB_8 exhibited a composition similar to swab seawater samples, characterized by a large proportion of *Candidatus Pelagibacter* and *uncultured bacteria*, along with *Roseomonas mucosa*, which was found exclusively in these samples (Fig. 4b). Several potential pathogens were identified as unique to specific sample origins. *Candidatus Babela*, *Candidatus Xiphinematobacter*, *Ewingella americana*, *Treponema vincentii* and *Vibrio splendidus* were identified as unique putative pathogens in the blow samples of *G. macrorhynchus. Microcystis aeruginosa* was found exclusively in the seawater samples collected with the swab, while nine unique potential pathogens were identified in filtered seawater: *Acidaminococcus intestini*, *Bacteroides caccae*, *Empedobacter brevis*, *Janibacter melonis*, *Laribacter hongkongensis*, *Mycobacterium abscessus*, *Prevotella paludivivens*, *Rickettsiales bacterium* and *Veillonella parvula*. Of the 30 putative pathogens identified in the blow samples of *G. macrorhynchus*, 14 were shared with the swab seawater samples, and 23 were common with the filtered seawater samples.

Within the EBC samples of *D. delphis*, nine potential pathogens were exclusively identified: *Achromobacter xylosoxidans*, *Bacillus licheniformis*, *Clostridium paraputrificum*, *Francisella philomiragia*, *Mycobacterium tuberculosis*, *Psychrobacter phenylpyruvicu*s, *Sphingomonas lutea*, *Xanthomonas axonopodis* and *uncultured Rhodocyclaceae*.

### Community diversity of putative pathogens

**Fig. 5.**
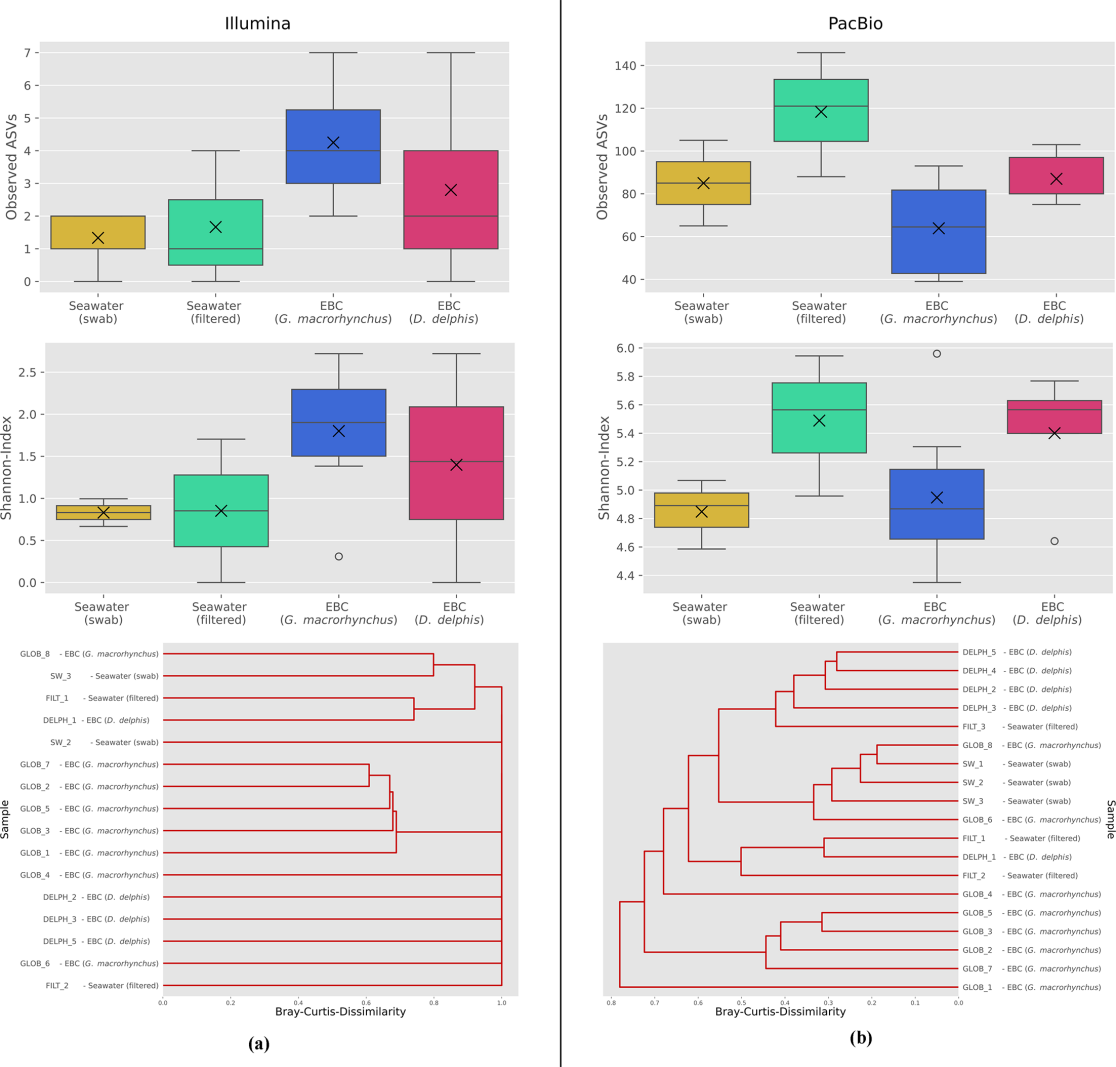
Boxplot diagram showing the alpha diversity for putative pathogens (top part) grouped according to their origin sequenced with Illumina (**a**) and PacBio (**b**). The alpha diversity was plotted on the y-axis using the number of observed ASVs and their values obtained by the calculation with the Shannon index. The mean value of the respective boxplots is labelled ‘X’. Dendrogram showing the hierarchical clustering of samples (bottom part) sequenced with Illumina (**a**) and PacBio (**b**) based on Bray–Curtis dissimilarity of their potential pathogen community composition (indicated on the x-axis). The respective samples and their origin are shown on the y-axis.

Alpha diversity of the potential pathogen community across the different sample origins was calculated using the same approach as for the prokaryotic community diversity, including the metrics observed ASVs and Shannon index. The analysis was based on 46 putative pathogen taxa identified through Illumina sequencing and 614 taxa identified through PacBio sequencing. The low number of putative pathogens in the Illumina dataset is reflected in the relatively low number of observed ASVs and Shannon index values (Fig. 5a, top part). Compared to PacBio, Illumina reported significantly less diversity (Mann−Whitney U test, p-value <0.05, Table S15). Filtered water samples reported the highest α diversity of potential pathogens in the PacBio dataset. Despite *α*-diversity of the potential pathogen community tending to be higher in EBC samples from *D. delphis* compared to those from *G. macrorhynchus* and the EBC samples from *D. delphis* exhibited lower variability than those from *G. macrorhynchus* (Fig. 5b, top part), there was no significant difference between sample origins (Mann−Whitney U test, p-value <0.05, Table S12).

*β*-Diversity of the putative pathogen community was assessed using the same approach as for the general microbial composition, based on Bray–Curtis dissimilarity. Analysis of the Illumina dataset revealed nine distinct branches, seven of which each contained only a single sample with a dissimilarity value of 1.0 compared to all other samples (Fig. 5a, bottom part). Clustering was observed among five of the eight EBC samples from *G. macrorhynchus*, as well as among samples GLOB_8, SW_3, FILT_1 and DELPH_1. Samples SW_1 (seawater swab), DELPH_4 (*D. delphis*) and FILT_3 (filtered seawater) were excluded from the Illumina analysis due to the absence of detectable potential pathogens (Fig. 4). The ANOSIM reported a positively significant grouping (*R*=0.247, p-value<0.05, Table S16).

The dendrogram constructed on the *β*-diversity values of the sample sequenced with PacBio data revealed six branches (Fig. 5b, bottom part), which, similar to the *β*-diversity analysis of the general microbial community (Fig. 3b, bottom part), corresponded to the respective sample origins. The first cluster comprised the four EBC samples from *D. delphis* (DELPH_2 to _5), along with the filtered seawater sample FILT_3. The swab seawater samples formed the second cluster together with the two *G. macrorhynchus* EBC samples GLOB_6 and GLOB_8. The third cluster included the remaining two filtered seawater samples (FILT_1 and FILT_2) along with the remaining *D. delphis* EBC sample (DELPH_1). The final three branches consisted of the remaining EBC samples from *G. macrorhynchus*. Among them, samples GLOB_4 and GLOB_1 showed a greater degree of dissimilarity compared to the others and therefore formed separate, individual branches, while the remaining *G. macrorhynchus* samples clustered together. As per the Illumina dataset, ANOSIM resulted in significant positive grouping (*R*=0.33, p-value<0.05, Table S17).

## Discussion

### Sample temporal and spatial variation

The two cetaceans species described in this study (*G. macrorhynchus* and *D. delphis*) were sampled three years apart and in two distant geographical locations (Fig. 1). Although it is not the scope of this work to characterize this temporal and spatial variation, it is important to address its possible influences on the presented results. Previous studies of the cetacean EBC microbiome have reported intraspecific geographical differences, depending on population location [5]. Therefore, the detected variations between *G. macrorhynchus* and *D. delphis* EBC microbiome might have been increased by the different location of the two sampled populations. From a temporal perspective, a previous study has reported cetacean EBC microbial community remaining stable over time [35] and therefore is questionable whether this effect might increase as well the difference detected between the two cetaceans species or not. However, in the case where temporal and spatial variation were strongly influencing the detected differences, the *β*-diversity analysis would have returned clusters based on either the year or the position and this was clearly not the case (Fig. 5a, b). Finally, temporal and spatial variation are not expected to influence neither the comparison between sequencing platforms nor the effectiveness of the detection of potential pathogens. In the first case, the comparison between platforms is performed on the same biological samples, with the variations being present in both datasets (Illumina and PacBio), hence not affecting the comparison. In the second case, the effectiveness of the detection of potential pathogen is heavily dependent on the database composition (MBPD) and the organism investigated (cetacean) rather than spatial or temporal variation in the data.

### Characterization of cetacean respiratory tract microbiota in EBC samples

The characterization of the respiratory microbiome in cetaceans is not recent, with the first study being conducted in 1989 [36]. Since then, research has advanced on the characterization of cetacean respiratory microbiomes targeting different species [7,12, 14, 15, 20, 37]. Previous studies analysing the bacterial community in the blow of *G. macrorhynchus* at the phylum level revealed a high prevalence of *Proteobacteria* [20], a pattern that was also observed in the blow samples of this study, particularly in those sequenced with PacBio (Fig. 2). *Firmicutes* on the other hand, previously identified as part of the core microbiome [20], was found only in small amounts in the EBC samples of *G. macrorhynchus* analysed with PacBio. In contrast, Illumina sequencing revealed *Bacteroidota* as the most abundant phylum, followed by *Proteobacteria* and *Actinobacteriota* (Fig. 2). However, all these phyla were also found in the seawater samples, which served as a comparison for the EBC samples of *G. macrorhynchus* and could therefore be distinguished from them.

To date, few articles have sought to sample and characterize the blow microbiota in free-ranging oceanic dolphins [7,9, 12, 20], compared to baleen whales [5,6, 10, 11, 13,15]. Among these, none have provided a characterization for *D. delphis*, making this study the first representation of the respiratory tract microbiota of this species from the analysis of EBC sample analysis. As demonstrated by Robinson and Nuuttila [21], successfully sampling this biological matrix in free-ranging dolphins, specifically the retention of quantifiable DNA concentrations, is highly challenging, which complicates its use for various research purposes. Particularly challenging are small dolphins travelling in close groups, whose fast movements and small blows make collecting EBC at the individual level very difficult.

Four of the five EBC samples of *D. delphis* showed a consistently high proportion of *Acidobacteria* (genus *Vicinamibacteraceae*), *Actinobacteria* (genus CL500) and *Chloroflexi* (SL56 marine group) with respect to the other samples from seawater and from *G. macrorhynchus* (Figs 2 and S2). The similarity and evenness of these four samples are particularly notable in the PacBio dataset, where alpha diversity showed minimal variation and the *β*-diversity revealed clear clustering (Fig. 3a, b). Additionally, one of the Illumina-sequenced samples identified an exclusive genus (*Suctoria*) part of the phylum *Ciliophora* in the EBC samples of *D. delphis* (Figs 2 and S2).

The fifth sample (DELPH_1), which had contact with seawater during sampling, exhibited a distinct microbial composition. Considering this contamination was known at the sampling stage, we expected DELPH_1 to be more like the filtered seawater samples (FILT_1, FILT_2 and FILT_3) than to the other EBC samples from *D. delphis*. *β*-diversity clustering confirmed our expectation reporting DELPH_1 in a cluster with filtered seawater samples rather than with the *D. delphis* ones (Fig. 3b, bottom part). This is important for several reasons: (i) indicates great potential of microbial community analysis for the monitoring of cetacean health, (ii) highlights how *D. delphis* and seawater microbial communities are different and (iii) how it is possible to separate samples based only on microbial community information. Additionally, a particular putative pathogen, *C. cynodegmi,* is found in high abundance in all the samples of this cluster. This organism is a relatively uncommon bacterial pathogen with low virulence compared to other members of its genus [38]. To the best of our knowledge, this is the first time that *C. cynodegmi* has been associated with cetaceans and its association is probably the result of the aforementioned contamination with seawater.

### Comparison of cetacean blow microbiota across hemispheres

Our analysis of cetacean blow microbiota reveals both shared and distinct bacterial communities when compared to recent findings from the Southern Hemisphere [18]. In this review, common bacterial genera in the blow microbiota of *Megaptera novaeangliae* were reported to be *Tenacibaculum*, *Pseudomonas*, *Leptotrichia* and *Corynebacteria* [18]. Two of these genera, *Tenacibaculum* and *Pseudomonas*, have been detected in *G. macrorhynchus* that was sampled in the Northern Hemisphere (Fig. 1). On the other hand, *Leptotrichia* was found only in the filtered seawater and *Corynebacteria* in all samples (Fig. S2). Potential pathogens reported in *M. novaeangliae* [18], specifically *Balneatrix*, *Staphylococcus* and *Streptococcus*, were not found in any of the samples, while Bacillia and *Clostridia* were found in low abundance in filtered seawater and in *D. delphis*. No specific patterns across hemisphere were detected, neither in the overall microbial community nor in the putative pathogens.

### Comparative analysis of Illumina and PacBio sequencing results

Marine investigation is often limited by funding, especially when long-term monitoring is needed [39]. Therefore, a balance between available budget and quality of the results yielded is critical to ensure a scientifically meaningful and economically feasible monitoring programme [39]. For this reason, comparative studies are essential to understand what the different methodologies can provide. In this study, we compare EBC from *D. delphis* and *G. macrorhynchus*, along with seawater samples from the same location as the latter, that were collected and sequenced using Illumina and PacBio platforms. These two platforms have different costs and provide different results even targeting the same gene, 16S rRNA in this case. PacBio offers the possibility to cover the entire length of the gene due to the long-read technology [40]. Illumina covers only a set of hypervariable regions of this gene, but at a lower cost than PacBio [41].

The two platforms yielded considerably different results as 350 and 2373 ASVs were identified with Illumina and PacBio sequencing, respectively. This disparity was also reflected in the number of identified phyla, 17 vs. 30, and potential pathogens, 18 vs. 46, respectively for Illumina and PacBio platforms. The number of ASVs from PacBio data was 6.78 times the Illumina, the increase in phyla was 1.76 times and 2.5 times in detecting putative pathogens. It is important to consider that the Illumina dataset focused solely on the hypervariable V4–V5 region of the 16S rRNA gene, while PacBio sequenced the full length of the 16S rRNA gene. Longer reads produced by platforms like PacBio have already been reported to improve taxonomic assignment and phylogenetic analysis [23,42,44]. Future investigations of cetacean respiratory tract microbiota may benefit from the use of long-read sequencing platforms, as these enable full-length coverage of the 16S rRNA gene. Such an approach is particularly advantageous when higher taxonomic resolution at lower classification levels is required.

Discrepancy between the two sequencing platforms was also evident in the alpha diversity metrics. Specifically, PacBio not only detected more ASVs but also yielded higher Shannon index values for both the general microbial community (Fig. 3b, top part) and the potential pathogen subset (Fig. 5b, top part), compared to Illumina. *α*-Diversity metrics reported significant differences in both the overall microbial community and the putative pathogens with Illumina capturing less diversity than PacBio (Tables S10 and S15). Moreover, the *β*-diversity analysis of the PacBio data fit well with the metadata of the samples, such as the origin of the samples, while Illumina does not detect meaningful clusters (Fig. 3, bottom part) despite ANOSIM reported similar significant R values for both sequencing platforms (Tables S8–S9 and S16–S17). Based on the Illumina results, no clear clustering could be defined (Fig. 3a, bottom part). Furthermore, the reliability of the Illumina dendrogram is also questionable, considering the great difference in Bray–Curtis dissimilarity values compared to the ones obtained in PacBio. Here, five distinct clusters were identified, with four largely corresponding to their respective sample origins (Fig. 3b, bottom part).

In the case of the putative pathogen community, the difference is even more pronounced. While the dendrogram, based on the PacBio data, showed strong similarities to the dendrogram of the general microbial community and therefore also reflected the sample origins (Figs 3b and 5b, bottom part), seven of the nine branches in the Illumina dendrogram represented stand-alone samples, with no apparent meaningful clustering (Fig. 5a, bottom part). This discrepancy may be due to the substantially lower number of potential pathogens identified in the Illumina dataset, with three samples yielding no detectable putative pathogens at all (Fig. 4A).

### Detection of potential pathogens in EBC samples

A review of the current state of the art in exploring EBC samples as a biological matrix for assessing the health status of cetacean populations highlights a significant methodological gap [45,46]. Until the publication of this study, it was not possible to achieve a detailed taxonomic resolution of the putative pathogens present in the microbial communities of the cetacean respiratory tract beyond the genus level. In some cases, studies only referenced the potential for pathogenicity [9,14, 20]. While previous results have provided valuable insights into topics such as the impact of sociality [15,37], interspecific differences in microbial profiles [15,20] and between geographic variation [14]. However, the inability to resolve potential pathogens at a finer taxonomic level represents a major knowledge gap in the identification of potential causes of diseases within these populations. Therefore, this study sought to advance this analysis by employing an innovative approach.

The pipeline ‘Multiple Bacterial Pathogen Detection’ (MBPD) was employed for the successful identification of potential pathogens. Originating from the silva database, the MBPD pipeline encompasses 1986 pathogenic taxa, of which 48.9% are associated with animal hosts [34].

Illumina-based sequencing identified 18 distinct putative pathogens, whereas PacBio sequencing resulted in the detection of 55 potential pathogens. This discrepancy, alongside the markedly different number of ASVs generated by each platform (409 vs. 2,647), may be attributed to the pipeline’s reliance on full-length 16S rRNA gene sequences for pathogen identification. Notably, *Mycoplasma* spp. cannot be reliably assigned using the V4–V5 region, a limitation particularly relevant to short-read platforms [34]. Further constraints are acknowledged by the pipeline developers, who emphasize that short-read sequencing (e.g. Illumina) may compromise taxonomic resolution, whereas long-read sequencing (e.g. PacBio) shows greater reliability, at least up to the genus level [23,34]. To validate taxonomic assignments in this study, classifications generated via MBPD were cross-referenced with the silva taxonomy at lower taxonomic ranks (data not shown). Since the database has been uploaded four years ago (https://github.com/LorMeBioAI/MBPD/tree/main/db), the taxonomy used is not updated to the current one used by silva. This caused incongruences due to differences between the current nomenclature and the previous one. The resources on which the MBPD database was built can be found in Table S1 of [34]. At present, this study is the first using MBPD to identify putative pathogens in cetaceans. Previous studies focused on soil microbiome [47], while the only application found in higher organisms is a recent study about the gut microbiome of pandas [48].

Beyond the difference in the number of identified potential pathogens, the PacBio data also revealed a higher relative proportion of putative pathogens within the samples than the Illumina data. A large proportion of putative pathogens identified in the Illumina dataset consisted of uncultured candidates, such as *uncultured bacterium*, *uncultured Rhodocyclaceae* or *uncultured Burkholderiales*. Some micro-organisms identified as potential pathogens, such as *Candidatus Pelagibacter*, are common and widely distributed marine bacteria [43,44, 49], which had already been detected in control seawater samples [12]. *Candidatus Pelagibacter* was found across all sample origins on both sequencing platforms, suggesting the possibility of cross-contamination during sample collection.

Despite the differences observed in putative pathogen detection between sequencing methods, the distribution of some potential pathogens, such as *T. dicentrarchi* and *R. denitrificans*, remained consistent across the EBC samples of *G. macrorhynchus. T. dicentrarchi* is known to be pathogenic in fish, causing fatal skin lesions in different species [50,51]. Apprill and colleagues [5] reported the frequent presence of unidentified *Tenacibaculum* species in the skin of healthy cetaceans, suggesting that members of this genus are unlikely to cause any disease in marine mammals. However, there are currently no studies analysing the effects of *Tenacibaculum* spp. at the species level in any cetacean species. *R. denitrificans*, on the other hand, has been previously associated with pathogenic capacity [52] in relation to its ability to perform nitric oxide reduction but its influence on cetacean health is still unknown. *R. denitrificans* is an aerobic anoxygenic phototrophic bacterium [53] found in high abundance in both PacBio- and Illumina-sequenced *G. macrorhynchus* blow samples, and this genus is frequently present in marine environments [54]. The relative abundance of these putative pathogens, along with uncultured *Candidatus*, was particularly high in the EBC samples GLOB_2, GLOB_3, GLOB_5 and GLOB_7 (Fig. 4B). This pattern was further reflected in the clustering of these samples in the potential pathogen dendrogram generated from both sequencing methods (Fig. 5, bottom part). A similar clustering pattern was observed in the dendrogram representing the general microbial community (Fig. 3, bottom part). A comparison of the two PacBio-based dendrograms revealed additional consistent clustering patterns, such as for the EBC samples derived from *D. delphis* and the filtered seawater samples. In the case of samples GLOB_6 and GLOB_8, which clustered with the swab seawater samples due to a similar putative pathogen composition, we found even identical clusterings across both dendrograms. These observations lead to the following conclusions: (i) the similarity in the composition of potential pathogens among samples is reflected in their clustering patterns, (ii) as seen in the overall microbial dendrogram, the composition of putative pathogens appears to be influenced by the sample origin and potential contamination (e.g. DELPH_1), and (iii) clustering based on the PacBio dataset seems to yield a more meaningful result considering the metadata.

Even though relative abundance and clustering appear to indicate a different pattern, most putative pathogens detected in the EBC samples were also present in seawater samples. However, a subset of potential pathogens was exclusively identified in the EBC samples. Thus, Illumina sequencing revealed seven such exclusive taxa for G. *macrorhynchus*, of which only *Candidatus Accumulibacter* retained its exclusivity when compared to the PacBio dataset. Conversely, *Candidatus Babela*, *Candidatus Xiphinematobacter*, *Ewingella americana*, *Treponema vincentii* and *Vibrio splendidus* were uniquely detected by PacBio sequencing and were not identified through Illumina-based analysis. Although their impacts on cetaceans are unknown, the *Vibrio* genus is usually associated with a variety of diseases in species of this taxonomic group [53,55] and *Treponema* spp. has been associated with ulcerative bacterial glossitis in *Stenella frontalis* [56]. Due to their similar microbial composition, all samples from *G. macrorhynchus*, except for GLOB_6 and GLOB_8, formed a distinct cluster based on the PacBio data. Interestingly, these two samples exhibited a profile of putative pathogens closely resembling that of the swab seawater samples, characterized by a high relative abundance of *Candidatus Pelagibacter* and uncultured bacteria, as well as the exclusive presence of *R. mucosa*. This distinct composition of the potential pathogens, reflected in the clustering of these samples with each other (Fig. 5B, bottom part), may indicate contamination of the samples with seawater.

In the EBC samples of *D. delphis*, Illumina sequencing identified four exclusive putative pathogens, of which only *S. paucimobilis* remained exclusive when cross-referenced with the PacBio dataset. In contrast, PacBio sequencing revealed a broader range of unique potential pathogens, such as *A. xylosoxidans*, *B. licheniformis*, *C. paraputrificum*, *F. philomiragia*, *M. tuberculosis*, *P. phenylpyruvicus*, *S. lutea*, *X. axonopodis* and an *uncultured Rhodocyclaceae*, of which none were detected via Illumina. These bacteria have been associated with various diseases and infections in humans and animals [57,62]. *Psychrobacter* spp. have been detected in multiple wound infections in different cetacean species [63,64]. *Mycobacterium* spp., namely, *M. pinnipedii*, which was detected in one EBC sample (DELPH_4), is a known agent of tuberculosis in other marine mammals, such as pinnipeds [65]. Its species name reflects this association and suggests a potential risk for free-ranging cetacean species as well. Other uncultured bacteria were also found to be prominent in the two cetacean species analysed in this study, namely *Burkholderiales* spp., *Rhodocyclaceae* spp. and *Burkholderia*. Particularly, *Burkholderia pseudomallei* is a known causative agent of melioidosis, an emerging disease in humans and animals, and has been reported to cause mortality in *Tursiops truncatus* [66]. As for *Rhodocyclaceae*, although considered to play a role in stimulating the human immune system and inducing Kawasaki disease, an idiopathic systemic vasculitis that predominantly damages coronary arteries in children [67], no studies have investigated its pathogenicity in marine mammals. These findings underscore the importance of further developing pathogen detection tools and coupling them with studies that assess the actual health impacts of these micro-organisms on cetaceans.

## Conclusion

This study underscores the potential of EBC microbial community analysis as a tool for monitoring cetacean health. The methodological approach employed, centred on the MBPD database, demonstrates a promising, minimally invasive strategy for the detection of putative pathogens which may cause diseases in cetaceans. The results revealed substantially lower performance of Illumina (representing short-read sequencing) compared with PacBio (representing long-read sequencing) in terms of microbial community profiling, clustering resolution and pathogen detection capabilities. These platform-specific variations offer valuable insights for establishing methodological guidelines in future studies using EBC microbiome analysis as a non-invasive tool to assess the health status of wild cetacean populations. Distinct and species-specific respiratory microbiota were observed among the cetaceans studied, with sufficient resolution to identify instances of contamination from ambient seawater. Future investigations should focus on refining bioinformatic workflows and integrating complementary sampling methodologies. Moreover, enhanced characterization of respiratory potential pathogens will be critical for advancing current monitoring tools.

## Supplementary material

10.1099/mgen.0.001773 Supplementary Material 1.

## Abbreviations

ANOSIM: analysis of similarities
ASV: amplicon sequence variant
CCS: circular consensus sequencing
EBC: exhaled breath condensate
ENA: Eastern North Atlantic
MBPD: Multiple Bacterial Pathogen Detection
