# Supplementary material for: Mapping potential pathogen profiling in cetacean blow: comparative insights from sequencing technologies
Source: Microb Genom. 2026 Jul 8;12(7):001773. doi: 10.1099/mgen.0.001773 (PMC13344881; doi:10.1099/mgen.0.001773)

Supplementary material

of:

Mapping Potential Pathogen Profiling in Cetacean Blow: Comparative Insights from Sequencing Technologies

*Johann Frederick Jaitner, *Nicola Gambardella, Luís Afonso, Raul Valente, Maria Paola Tomasino, Ana Mafalda Correia, Massimiliano Rosso, Filipe Alves, Catarina Magalhães

Johann Frederick Jaitner and Nicola Gambardella have contributed equally to this work.

Supplementary Table 1: Shows for each sample, from left to right, its origin, collection method, species, date of sampling, location (latitude/longitude) and comments on the sampling proccess.

Sample-Code	Sample-Code (converted)	Sample Origin	Collection method	Species	Sampling day (dd/mm/yyyy)	Latitude	Longitude	Comments
**CK-SW-15**	**SW_1**	Seawater (swab)	Cotton Swab (SW)	-	09/11/2018	-	-	
**CK-SW-16**	**SW_2**	Seawater (swab)	Cotton Swab (SW)	-	09/11/2018	-	-	
**CK-SW-18**	**SW_3**	Seawater (swab)	Cotton Swab (SW)	-	30/11/2018	-	-	
**St-16-17**	**FILT_1**	Seawater (filtered)	SW 1L filtered with sterivex	-	09/11/2018	-	-	
**St-18**	**FILT_2**	Seawater (filtered)	SW 1L filtered with sterivex	-	30/11/2018	-	-	
**St-19-20-22**	**FILT_3**	Seawater (filtered)	SW 1L filtered with sterivex	-	12/12/2018	-	-	
**K-B-15A**	**GLOB_1**	EBC (*Globicephala macrorhynchus*)	pole + swab	*Globicephala macrorhynchus*	09/11/2018	32.5922	-16.873	
**K-B-16A**	**GLOB_2**	EBC (*Globicephala macrorhynchus*)	pole + swab	*Globicephala macrorhynchus*	09/11/2018	32.5922	-16.873	
**K-B-17A**	**GLOB_3**	EBC (*Globicephala macrorhynchus*)	pole + swab	*Globicephala macrorhynchus*	09/11/2018	32.5392	-16.933	
**K-B-18A**	**GLOB_4**	EBC (*Globicephala macrorhynchus*)	pole + swab	*Globicephala macrorhynchus*	30/11/2018	32.5940	-16.896	
**K-B-19A**	**GLOB_5**	EBC (*Globicephala macrorhynchus*)	pole + swab	*Globicephala macrorhynchus*	12/12/2018	32.5814	-16.8020	
**K-B-20A**	**GLOB_6**	EBC(*Globicephala macrorhynchus*)	pole + swab	*Globicephala macrorhynchus*	12/12/2018	32.5814	-16.8020	
**K-B-21A**	**GLOB_7**	EBC (*Globicephala macrorhynchus*)	pole + swab	*Globicephala macrorhynchus*	12/12/2018	32.5897	-16.8916	
**K-B-22A**	**GLOB_8**	EBC (*Globicephala macrorhynchus*)	pole + swab	*Globicephala macrorhynchus*	12/12/2018	32.5897	-16.8916	
**K-B-2021-1A**	**DELPH_1**	EBC (*Delphinus delphis*)	pole + swab	*Delphinus delphis*	01/09/2021	41.3652	-8.9443	The swab touched the water and the animal.
**K-B-2021-2A**	**DELPH_2**	EBC (*Delphinus delphis*)	pole + swab	*Delphinus delphis*	02/09/2021	41.3652	-8.9443	
**K-B-2021-3A**	**DELPH_3**	EBC (*Delphinus delphis*)	pole + swab	*Delphinus delphis*	03/09/2021	41.1896	-8.9153	
**K-B-2021-4A**	**DELPH_4**	EBC (*Delphinus delphis*)	pole + swab	*Delphinus delphis*	24/09/2021	41.2227	-8.9444	
**K-B-2021-5A**	**DELPH_5**	EBC (*Delphinus delphis*)	pole + swab	*Delphinus delphis*	24/09/2021	41.4831	-8.9004	

Supplementary Table 2: Shows the total number of ASVs and the number of different results at the different taxonomic levels based on the SILVA database (used for relative abundance at the phylum level). It also shows the number of pathogenic ASVs identified using the MDPD database (used for the relative abundance of pathogens). The data are displayed separately for samples sequenced with Illumina (left) and PacBio (right).

		**Illumina**	**PacBio**
**Total ASV**		**350**	**2373**
**SILVA**: Number of different results for the respective taxonomic level	**Domain**	3	1
	**Phylum**	17	30
	**Class**	26	63
	**Order**	52	147
	**Family**	70	242
	**Genus**	79	376
**Identified Pathogens**		**46**	**614**

Supplementary Table 3 - PacBio: DADA2 statistics related to the ASVs genetation. Table exported directly from QIIME2 after using qiime dada2 denoise-ccs command.

PACBIO	PACBIO	PACBIO	PACBIO	PACBIO	PACBIO	PACBIO	PACBIO	PACBIO
sample-id	input	primer-removed	percentage of input primer-removed	filtered	percentage of input passed filter	denoised	non-chimeric	percentage of input non-chimeric
#q2:types	numeric	numeric	numeric	numeric	numeric	numeric	numeric	numeric
CK_SW_15	19908	19783	99.37.00	14581	73.24.00	6822	6760	34.36.00
CK_SW_16	21223	21119	99.51.00	15666	74.22.00	8354	8236	39.21.00
CK_SW_18	16061	15994	99.58.00	11704	73.27.00	5431	5351	33.32.00
K_B_15A	6317	6303	100.18.00	5371	85.02.00	4588	4585	72.58.00
K_B_16A	11812	11763	99.59.00	9002	76.21.00	4885	4780	40.47.00
K_B_17A	10351	10302	99.53.00	7869	76.02.00	4245	4215	41.12.00
K_B_18A	9650	9603	99.51.00	6879	71.28.00	2729	2643	27.39.00
K_B_19A	11616	11551	99.44.00	8629	74.29.00	4205	4168	36.28.00
K_B_2021_1A	24613	24458	99.37.00	18140	73.7	9733	8540	34.7
K_B_2021_2A	23687	23571	99.51.00	17286	73.38.00	9289	9074	38.31.00
K_B_2021_3A	18053	17986	100.03.00	13387	74.15.00	6660	6314	35.37.00
K_B_2021_4A	22008	21913	99.57.00	16054	73.35.00	8280	8036	36.51.00
K_B_2021_5A	22651	22553	99.57.00	16725	74.24.00	8420	8164	36.04.00
K_B_20A	16765	16675	99.46.00	12021	71.7	5159	5133	31.02.00
K_B_21A	28045	27907	99.51.00	20219	72.09.00	6610	6482	23.11
K_B_22A	18915	18813	99.46.00	13777	73.24.00	6540	6510	34.42.00
St_13	20704	20631	100.05.00	15740	76.02.00	7712	7547	36.45.00
St_14	18344	18249	99.48.00	13646	74.39.00	5420	5397	29.42.00
St_16-17	46805	46555	99.47.00	35351	75.53.00	21807	19523	42.11.00
St_18	34688	34540	99.57.00	25458	73.39.00	10417	10099	29.11.00
St_192022	26702	26555	99.45.00	19986	75.25.00	12137	11397	43.08.00

Supplementary Table 3 - Illumina: DADA2 statistics related to the ASVs genetation. Table exported directly from QIIME2 after using qiime dada2 denoise-ccs command.

ILLUMINA	ILLUMINA	ILLUMINA	ILLUMINA	ILLUMINA	ILLUMINA	ILLUMINA	ILLUMINA	ILLUMINA
sample-id	input	filtered	percentage of input passed filter	denoised	merged	percentage of input merged	non-chimeric	percentage of input non-chimeric
#q2:types	numeric	numeric	numeric	numeric	numeric	numeric	numeric	numeric
CK-SW-15	8473	1767	21.25	1392	575	7.19	345	4.07
CK-SW-16	13958	3710	26.58.00	3226	1764	13.04	1045	7.49
CK-SW-18	12812	3087	24.09.00	2551	1511	12.19	703	5.49
K-B-15A	5583	1534	27.48.00	1256	814	14.58	670	12
K-B-16A	8735	2341	26.8	2025	1578	18.07	845	10.07
K-B-17A	8076	2445	30.27.00	2033	1330	16.47	678	8.4
K-B-18A	15359	3158	20.56	2523	1543	10.05	765	5.38
K-B-19A	24629	6118	25.24.00	5378	3477	14.12	1431	6.21
K-B-2021-1A	18245	5046	28.06.00	4541	3544	19.42	795	4.36
K-B-2021-2A	6550	1748	27.09.00	1578	990	15.11	413	6.31
K-B-2021-3A	6278	1752	28.31.00	1463	882	14.05	573	9.13
K-B-2021-4A	4946	1253	25.33.00	1076	619	12.52	306	6.19
K-B-2021-5A	35003	8920	25.48.00	8256	5658	16.16	2374	7.18
K-B-20A	18550	3996	21.54	3425	2206	12.29	922	5.37
K-B-21A	9636	2208	23.31	1427	778	8.07	392	4.07
K-B-22A	12270	3034	25.13.00	2619	1221	10.35	608	5.36
St-13	28236	7500	26.56.00	6686	4459	16.19	1885	7.08
St-14	19271	4610	24.32.00	3591	1764	9.15	1085	6.03
St-16-17	20008	4901	24.5	4508	3296	16.47	1187	6.33
St-18	7735	1841	23.8	1229	712	9.2	342	4.42
St-19-20-22	10310	2810	27.26.00	2541	1718	17.06	859	8.33

Supplementary Table 4: results for the Wilcoxon signed-rank test run on α diversity of the overall microbial community from the PacBio dataset

	W-val	alternative	p-val	RBC	CLES
Wilcoxon	0	two-sided	3.81e-06	1	NaN

Supplementary Table 5: results for the Mann-Whitney U test run on α diversity of the overall microbial community from the PacBio dataset

Contrast	A	B	U-val	alternative	p-unc	p-corr	p-adjust	hedges
Sample Origin	EBC (D. delphis)	EBC (G. macrorhynchus)	29	two-sided	0.22	0.42	fdr_bh	0.67
Sample Origin	EBC (D. delphis)	Seawater (filtered)	3	two-sided	0.25	0.42	fdr_bh	-1.12
Sample Origin	EBC (D. delphis)	Seawater (swab)	4	two-sided	0.39	0.47	fdr_bh	-0.59
Sample Origin	EBC (G. macrorhynchus)	Seawater (filtered)	3	two-sided	0.08	0.42	fdr_bh	-1.41
Sample Origin	EBC (G. macrorhynchus)	Seawater (swab)	6	two-sided	0.28	0.42	fdr_bh	-0.92
Sample Origin	Seawater (filtered)	Seawater (swab)	6	two-sided	0.7	0.7	fdr_bh	0.59

Supplementary Table 6: results for the Wilcoxon signed-rank test run on α diversity of the overall microbial community from the Illumina dataset

	W-val	alternative	p-val	RBC	CLES
Wilcoxon	0	two-sided	0.000213	1	NaN

Supplementary Table 7: results for the Mann- Whitney U test run on α diversity of the overall microbial community from the Illumina dataset

Contrast	A	B	U-val	alternative	p-unc	p-corr	p-adjust	hedges
Sample Origin	EBC (D. delphis)	EBC (G. macrorhynchus)	17.5	two-sided	0.77	1	fdr_bh	0.17
Sample Origin	EBC (D. delphis)	Seawater (filtered)	6	two-sided	0.86	1	fdr_bh	0.34
Sample Origin	EBC (D. delphis)	Seawater (swab)	8	two-sided	1	1	fdr_bh	0.37
Sample Origin	EBC (G. macrorhynchus)	Seawater (filtered)	10	two-sided	0.69	1	fdr_bh	0.48
Sample Origin	EBC (G. macrorhynchus)	Seawater (swab)	14.5	two-sided	0.68	1	fdr_bh	0.48
Sample Origin	Seawater (filtered)	Seawater (swab)	3	two-sided	1	1	fdr_bh	0

Supplementary Table 8: results for the analysis of similarities (ANOSIM) run on β diversity of the overall microbial community from the PacBio dataset

	ANOSIM results
method name	ANOSIM
test statistic name	R
sample size	19
number of groups	4
test statistic	0.366
p-value	0.011
number of permutations	999

Supplementary Table 9: results for the analysis of similarities (ANOSIM) run on β diversity of the overall microbial community from the Illumina dataset

	ANOSIM results
method name	ANOSIM
test statistic name	R
sample size	19
number of groups	4
test statistic	0.304
p-value	0.001
number of permutations	999

Supplementary Table 10: results for the Mann- Whitney U test run on α diversity of the overall microbial community from the Illumina dataset using “Sample Origin” and “Platfrom” (Pacbio or Illumina) as covariates

Contrast	Sample Origin	A	B	U-val	alternative	p-unc	p-corr	p-adjust	hedges
Sample Origin	-	EBC (D. delphis)	EBC (G. macrorhynchus)	86.5	less	0.64	0.73	fdr_bh	0.2
Sample Origin	-	EBC (D. delphis)	Seawater (filtered)	19	less	0.26	0.62	fdr_bh	-0.43
Sample Origin	-	EBC (D. delphis)	Seawater (swab)	27	less	0.4	0.62	fdr_bh	-0.06
Sample Origin	-	EBC (G. macrorhynchus)	Seawater (filtered)	29	less	0.19	0.62	fdr_bh	-0.68
Sample Origin	-	EBC (G. macrorhynchus)	Seawater (swab)	44.5	less	0.41	0.62	fdr_bh	-0.27
Sample Origin	-	Seawater (filtered)	Seawater (swab)	18	less	0.73	0.73	fdr_bh	0.32
Platform	-	Illumina	PacBio	0	less	0	0		-4.14
Sample Origin * Platform	EBC (D. delphis)	Illumina	PacBio	0	less	0	0.01	fdr_bh	-8.08
Sample Origin * Platform	EBC (G. macrorhynchus)	Illumina	PacBio	0	less	0	0	fdr_bh	-3.27
Sample Origin * Platform	Seawater (filtered)	Illumina	PacBio	0	less	0.1	0.1	fdr_bh	-2.99
Sample Origin * Platform	Seawater (swab)	Illumina	PacBio	0	less	0.05	0.07	fdr_bh	-7.12

Supplementary Table 11: results for the Wilcoxon signed-rank test run on α diversity of the potential pathogen community from the PacBio dataset

	W-val	alternative	p-val	RBC	CLES
Wilcoxon	0	two-sided	0.000143	1	NaN

Supplementary Table 12: results for the Mann-Whitney U test run on α diversity of the potential pathogen community from the PacBio dataset

Contrast	A	B	U-val	alternative	p-unc	p-corr	p-adjust	hedges
Sample Origin	EBC (D. delphis)	EBC (G. macrorhynchus)	31	two-sided	0.12	0.24	fdr_bh	1.15
Sample Origin	EBC (D. delphis)	Seawater (filtered)	2	two-sided	0.13	0.24	fdr_bh	-1.39
Sample Origin	EBC (D. delphis)	Seawater (swab)	7	two-sided	1	1	fdr_bh	0.11
Sample Origin	EBC (G. macrorhynchus)	Seawater (filtered)	1	two-sided	0.02	0.14	fdr_bh	-2.12
Sample Origin	EBC (G. macrorhynchus)	Seawater (swab)	5	two-sided	0.19	0.24	fdr_bh	-0.91
Sample Origin	Seawater (filtered)	Seawater (swab)	8	two-sided	0.2	0.24	fdr_bh	1.07

Supplementary Table 13: results for the Wilcoxon signed-rank test run on α diversity of the potential pathogen community from the Illumina dataset

	W-val	alternative	p-val	RBC	CLES
Wilcoxon	0	two-sided	0.000459	1	NaN

Supplementary Table 14: results for the Mann-Whitney U test run on α diversity of the potential pathogen community from the Illumina dataset

Contrast	A	B	U-val	alternative	p-unc	p-corr	p-adjust	hedges
Sample Origin	EBC (D. delphis)	EBC (G. macrorhynchus)	12	two-sided	0.27	0.5 4	fdr_bh	-0.63
Sample Origin	EBC (D. delphis)	Seawater (filtered)	9.5	two-sided	0.65	0.78	fdr_bh	0.38
Sample Origin	EBC (D. delphis)	Seawater (swab)	9.5	two-sided	0.64	0.78	fdr_bh	0.54
Sample Origin	EBC (G. macrorhynchus)	Seawater (filtered)	20	two-sided	0.12	0.36	fdr_bh	1.33
Sample Origin	EBC (G. macrorhynchus)	Seawater (swab)	23	two-sided	0.03	0.18	fdr_bh	1.70
Sample Origin	Seawater (filtered)	Seawater (swab)	4.5	two-sided	1	1	fdr_bh	0.16

Supplementary Table 15: results for the Mann-Whitney U test run on α diversity of the potential pathogen community from the Illumina dataset using “Sample Origin” and “Platfrom” (Pacbio or Illumina) as covariates.

Contrast	Sample Origin	A	B	U-val	alternative	p-unc	p-corr	p-adjust	hedges
Sample Origin	-	EBC (D. delphis)	EBC (G. macrorhynchus)	83	less	0.57	0.74	fdr_bh	0.27
Sample Origin	-	EBC (D. delphis)	Seawater (filtered)	26.5	less	0.37	0.74	fdr_bh	-0.27
Sample Origin	-	EBC (D. delphis)	Seawater (swab)	31.5	less	0.59	0.74	fdr_bh	0.04
Sample Origin	-	EBC (G. macrorhynchus)	Seawater (filtered)	45	less	0.43	0.74	fdr_bh	-0.56
Sample Origin	-	EBC (G. macrorhynchus)	Seawater (swab)	52	less	0.63	0.74	fdr_bh	-0.23
Sample Origin	-	Seawater (filtered)	Seawater (swab)	21.5	less	0.74	0.74	fdr_bh	0.27
Platform	-	Illumina	PacBio	0	less	0	0		-4.04
Sample Origin * Platform	EBC (D. delphis)	Illumina	PacBio	0	less	0.01	0.01	fdr_bh	-8.58
Sample Origin * Platform	EBC (G. macrorhynchus)	Illumina	PacBio	0	less	0	0	fdr_bh	-3.68
Sample Origin * Platform	Seawater (filtered)	Illumina	PacBio	0	less	0.05	0.05	fdr_bh	-4.53
Sample Origin * Platform	Seawater (swab)	Illumina	PacBio	0	less	0.04	0.05	fdr_bh	-4.73

Supplementary Table 16: results for the analysis of similarities (ANOSIM) run on β diversity of the potential pathogen community from the Illumina dataset

	ANOSIM results
method name	ANOSIM
test statistic name	R
sample size	16
number of groups	4
test statistic	0.247
p-value	0.01
number of permutations	999

Supplementary Table 17: results for the analysis of similarities (ANOSIM) run on β diversity of the potential pathogen community from the PacBio dataset

	ANOSIM results
method name	ANOSIM
test statistic name	R
sample size	19
number of groups	4
test statistic	0.33
p-value	0.014
number of permutations	999

Supplementary Figure 1: rarefaction curve for Illumina (top) and PacBio (bottom) reporting the sequencing depth for all samples included in the study.

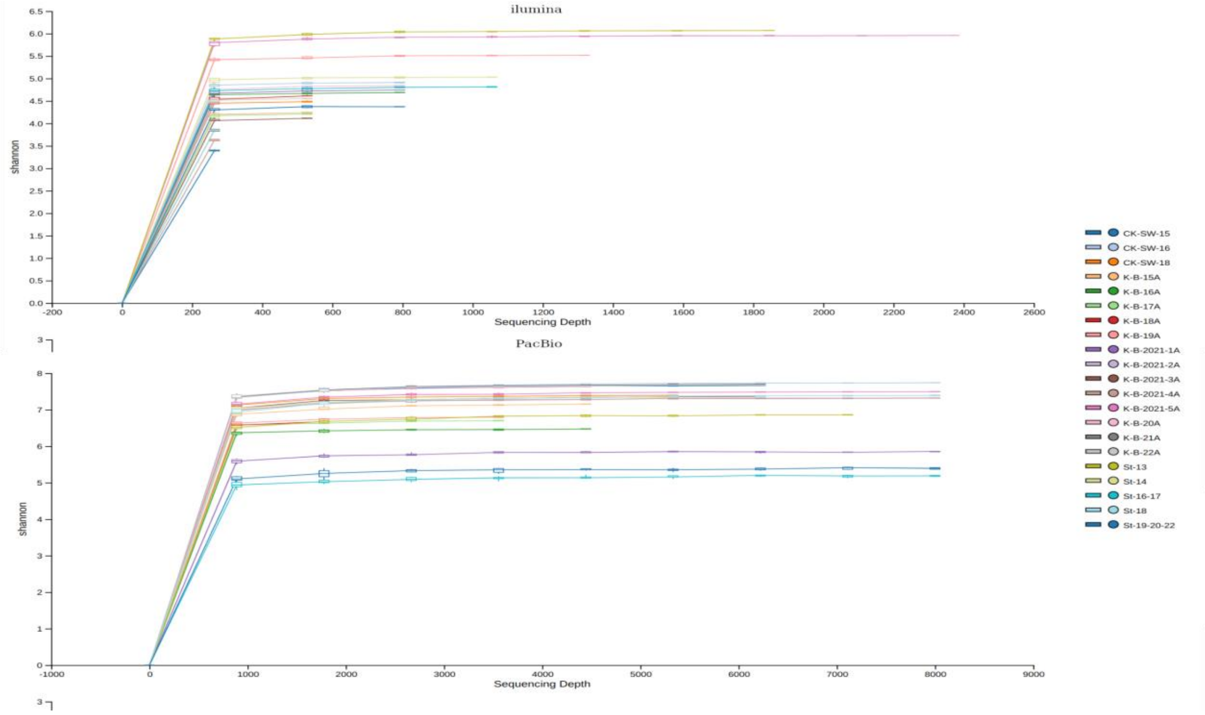

Supplementary Figure 2: Barplot showing the relative abundance of the Top 10 Genera from the samples sequenced with Illumina (A) and PacBio (B), both based on the SILVA database. The remaining genera were summed up under “Other”. Bars represent different samples, and they were grouped by their sample origin. The colored area represents the percentage of the respective genera in the legend from the entire sample, sorted according to their relative abundance across all samples.

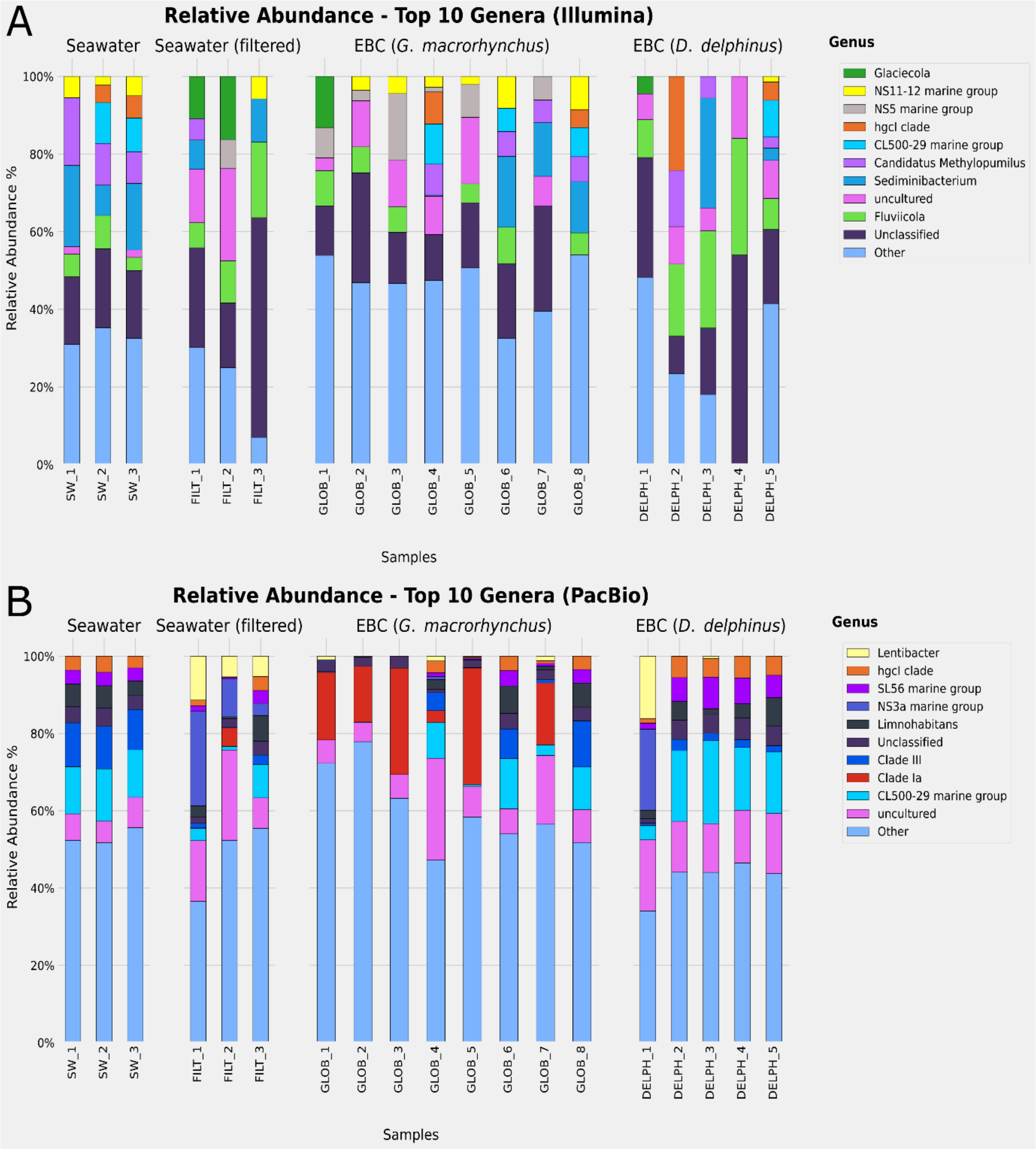

Supplement: Supplementary Material 1. [file mgen-12-01773-s001.pdf]
